# *WOX* genes expression during the formation of new lateral roots from secondary structures in *Populus nigra* (L.) taproot

**DOI:** 10.1038/s41598-020-75150-1

**Published:** 2020-11-03

**Authors:** Barbara Baesso, Mattia Terzaghi, Donato Chiatante, Gabriella Stefania Scippa, Antonio Montagnoli

**Affiliations:** 1 Department of Biotechnology and Life Science, University of Insubria, Via Dunant, 3, 21100 Varese, VA Italy; 2 Department of Chemistry and Biology ‘A. Zambelli’, University of Salerno, Via Giovanni Paolo II, 132, 84084 Fisciano, SA Italy; 3 Department of Biosciences and Territory, University of Molise, Contrada Fonte Lappone, 86090 Pesche, IS Italy

**Keywords:** Biological techniques, Molecular biology, Plant sciences

## Abstract

Despite the large amounts of data available on lateral root formation, little is known about their initiation from secondary structures. In the present work, we applied a bending treatment to *Populus nigra* (L.) woody taproots to induce the formation of new lateral roots. The development of lateral roots was monitored by stereomicroscopic examination of cross-sections. Tissues were sampled from the bending zone in the proximity of the vascular cambium before (time 0) and after the application of bending at three different time points (24, 48, and 72 h) and analyzed for the expression of *P. nigra* WOX homologs. The initiation of new lateral roots was observed to originate from the vascular cambium zone and was followed by primordium formation and root emergence. *PnWOX4a*, *PnWOX4b*, *PnWOX5a*, *PnWOX5b*, *PnWOX11/12a*, and *PnWOX11/12b* were shown to be expressed during the formation of new lateral roots at different developmental stages. The mechanical stress simulated by bending treatment was shown to activate the molecular mechanism leading to the expression of WOX genes, which are hypothesized to control SLR formation in the cambium zone of poplar taproot.

## Introduction

Many studies have demonstrated that WUSCHEL-RELATED HOMEOBOX (*WOX*) transcription factors are involved in controlling the early stages of embryogenesis, the maintenance of meristem activity, and the formation of lateral organs, such as lateral roots, leaves, and floral primordia^1,2^. Moreover, it has been reported that the action of *WOX*s is regulated by CLE peptides (CLAVATA3/EMBRYO SURROUNDING REGION-RELATED)^3^ and by the two major plant hormones: (1) auxins in the root apical meristem (RAM) and the cambium, and (2) cytokinins in the shoot apical meristem (SAM)^4^.

In recent years, the molecular events leading to the initiation and development of both lateral (LR) and adventitious roots (AR) from primary tissues have been extensively investigated^5,6^. In *Arabidopsis thaliana, WOX5* acts downstream of SHORTROOT (SHR)/SCARECROW (*SCR*) genes in maintaining the stem cell identity (homeostasis) of the root apical meristem (RAM) quiescent center^7^. In addition, *AtWOX5* is involved in the formation of LR and AR primordia as well as in root regeneration^8,9^. The expression of two homologs of *AtWOX5*, *PtWOX5a* and *PtWOX5b*, was also found in the RAM of *Populus tomentosa*^10^. Moreover, as *PtWOX5a* is specifically expressed in tips of adventitious and lateral roots, it has been proposed to be involved in AR development^11^. *WOX11* and *WOX12*, whose expression in *A. thaliana* has been related to both callus and AR formation, are directly regulated by the auxin-signaling pathway^12^. Additionally, *WOX11* seems to promote activation of *WOX5/7* during the transition from root founder cells to root primordia^13^. High expression of two homologs of *AtWOX11* and *AtWOX12* was found in *P. tomentosa* (*PtWOX11/12a* and *PtWOX11/12b*) and in the hybrid *P. deltoides* × *P. euramericana* (*PeWOX11a* and *PeWOX11b*) during AR formation^14^. *WOX4* plays a crucial role in maintaining vascular cambium organization during secondary growth^15,16^. In *A. thaliana, WOX4* seems to promote procambial and cambial activity in both roots and shoots, where it is expressed in the cambium^17^. The two homologs *WOX4a* and *WOX4b* in *Populus* are expressed in the vascular cambium, where they seem to control cell division activity^18^. High levels of *WOX4* expression were found during AR formation in *P. tomentosa*^10^.

In root secondary structures, new secondary lateral roots (SLRs) are continuously produced in response to environmental stimuli (i.e. variations of ion concentration in the soil matrix or mechanical stresses)^19,20^. Nevertheless, still little is known about the tissue origin of the initial cells that give rise to a new root primordium, and the molecular mechanism involved in the formation and development of new SLRs. To counter this lack of knowledge, studies in woody species have been focused on: (a) the mechanism that regulates the development of a specific root system architecture (RSA) and (b) how this RSA may be subject to ongoing variations^21^. The few anatomical studies carried out so far demonstrated the involvement of the vascular cambium during SLRs formation in different woody^20,22,23^ and non-woody^24^ plant species. Furthermore, a recent review suggested that only stem cells of the vascular cambium derived from pericycle cells positioned opposite xylem poles may maintain the competence to become founder cells of new SLRs^25^.

The two-fold aim of the present study was to investigate, at both the anatomical and molecular level, (i) the tissue originating SLRs in taproot characterized by secondary structure and (ii) if *WOX* genes could play a role during SLR formation and development in Poplar. We hypothesized that if *WOX* genes are expressed in meristematic tissues and primary structures during lateral root formation, then they might be involved in new lateral root formation of secondary structures. To test our hypothesis, we firstly induced SLR formation in *Populus nigra* (L.) taproot by the application of mechanical stress, and then investigated the anatomical traits of cross-sections together with the expression of different *WOX* genes (*PnWOX*s), through RT-PCR, at four different developmental stages.

## Results

### Anatomical features

**Figure 1:**
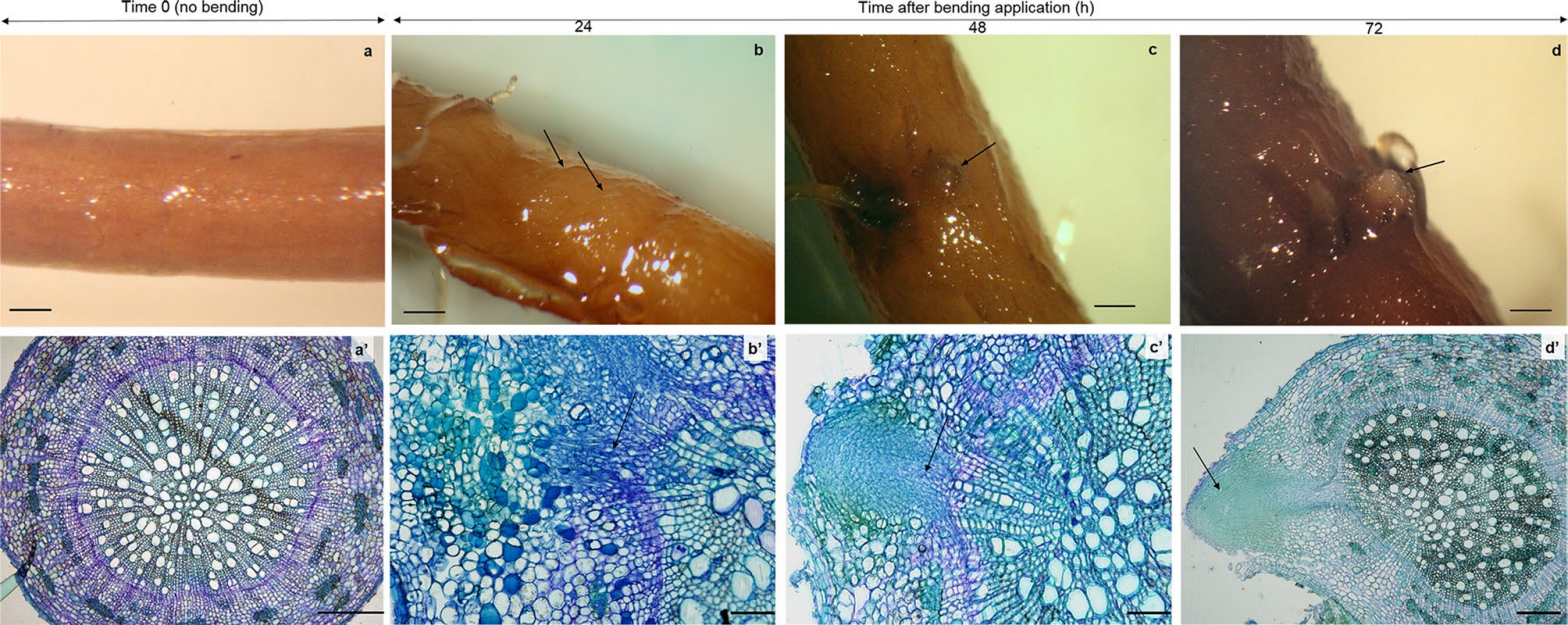
Cross-sections of SLR formation zones in poplar taproot at different time points. (**a**–**a**′) time point 0 when no lateral root primordia initiation is observed; (**b**–**b**′) 24 h after bending application when intensive cell division activity is visible; (**c**–**c**′) 48 h after bending application when vascular connections start to form in the vascular zone. (**d**–**d**′) 72 h after bending application when the lateral root primordium emerges from the taproot. Bars = 1 mm for panels **a**–**b**–**c**–**d**; Bars = 200 μm for panels **a**′–**b**′–**c**′–**d**′.

The observation of transverse sections of taproot secondary structure was performed on 15 seedlings before bending (time-point 0; Fig. 1a, a′) and for each of the three-time points following bending application (Fig. 1b and b′, c and c′, d and d′). The analysis showed that the system used for applying bending treatment homogeneously triggered the development of lateral root formation for each of the time-points considered and across all 15 samples analyzed. Therefore, anatomical features presented in Fig. 1 were observed for all samples analyzed.

Twenty-four hours after the application of bending, several swellings were detectable on the taproot surface (Fig. 1b). Anatomical observations revealed that in correspondence of these swellings numerous cells are dividing in the vascular cambium zone (Fig. 1b′). Thus, although in the present work it was not possible to identify the exact moment of founder cell specification, the formation of a new lateral root primordium was observed (Fig. 1b′). At the second time point (48 h), bump formation (Fig. 1c) occurred at the taproot surface and differentiation of vascular connections to the new lateral root primordia was observed in the cross section (Fig. 1c′). Finally, 72 h after bending application, protrusion of the new lateral root was observed (Fig. 1d, d′).

### *WOX* genes expression

**Figure 2:**
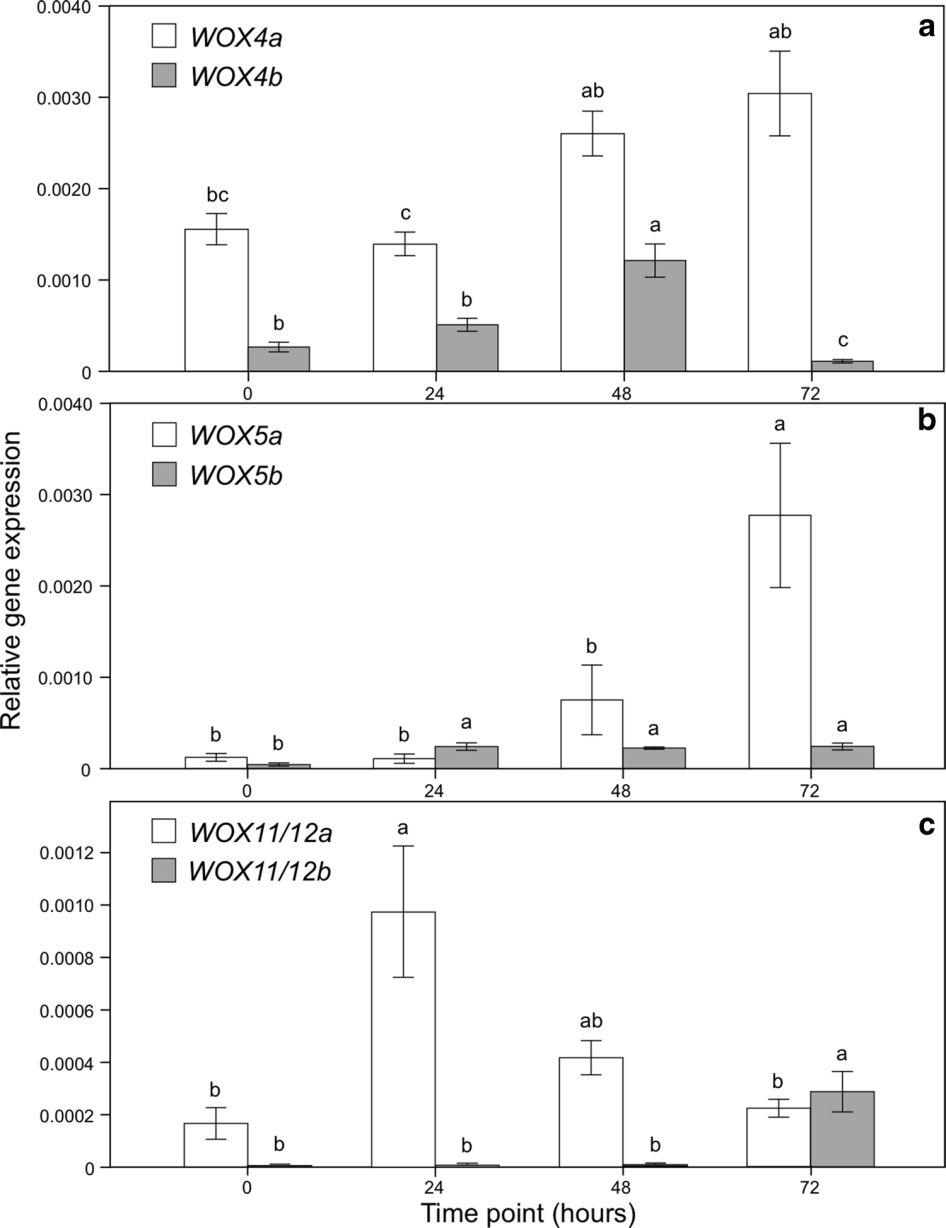
Quantitative expression analysis of *WOX4a* and *WOX4b* (**a**), *WOX5a* and *WOX5b* (**b**), *WOX11/12a* and *WOX11/12b* (**c**) at different time points. Data represent the mean of 15 independent replicates ± SE. Statistical differences (*p* < 0.05) are indicated by different letters (Bonferroni test).

To analyze the expression of *P. nigra* WOX homologues through RT-PCR, root tissues were sampled from the bending zone in the proximity of the vascular cambium before (time 0) and after the application of bending at three different time points (24, 48, and 72 h). At the first time point after bending application (24 h), when cambium cell division takes place, genes *PnWOX4a, PnWOX4b, PnWOX5a,* and *PnWOX11/12b* did not show any significant change in expression compared to taproot before bending (Time 0), while on the contrary *WOX11/12a* and *WOX 5b* reached their maximum expression (Fig. 2). In particular, the expression of *WOX11/12a* peaked at 24 h after bending application and then decreased (Fig. 2c). *PnWOX5b* was already induced at the first time point after bending application and its expression remained constant afterwards (Fig. 2b). At the second time point (48 h), expression of *PnWOX4a* and *PnWOX4b* showed a significant increase to a maximum level (Fig. 2a), suggesting the involvement of these genes in the differentiation of vascular vessels connecting new SLR primordia to the cambial tissue. Both *PnWOX5a* and *WOX11/12b* expression significantly peaked at the later stage of new SLR emergence, 72 h after bending application (Fig. 2b, c).

## Discussion

Here we present a short report showing the results of molecular and anatomical investigations aimed at improving our understanding of the mechanisms involved in lateral root formation from secondary tissues in *Populus nigra*. The application of bending to Poplar taproot to mimic mechanical stress occurring in nature successfully induced the formation of new lateral roots in secondary structure as already observed in previous works^22,24^.

Interestingly, expression of all *WOX* genes analyzed increased during the formation and development of SLR. However, our data revealed a complex and differential timing in their expression during the four steps considered. Therefore, findings presented here highlighted the importance of considering *WOX* genes during the formation of lateral roots from a secondary structure in Poplar. In particular, 24 h after bending application, when anatomical analysis revealed the occurrence of cell division in the vascular cambium zone, in correspondence of the swelling on the root surface, both *WOX5b* and *WOX11/12a* reached their maximum expression. These findings are in line with previous studies that showed the involvement of *WOX11/12a* in cell division in root meristems during the first steps of root development both in primary^10,12–14,26,27^ and secondary^24^ structure. Of relevant importance to our finding are the works of Liu et al.^10^ and Liu et al.^12^ which suggest that the combined action of *WOX11* and *12* mediates the transition of procambium stem cells to root founder cells during AR development, and showed that *WOX5b* is expressed in the RAM during AR regeneration in poplar.

The second time-point, 48 h after bending application, was a crucial step in SLR formation, since the cross-section analysis revealed differentiation of vascular connections to new lateral root primordia. At this time-point, both *WOX4a* and *4b* genes reached their maximum expression, hinting at a possible involvement of these genes in connecting SLRs to the parental root. In support of this hypothesis, we found that in the literature *WOX4* is reported as a regulator of cambium cell activity/inactivity, and identity^15,18,28^, and is up-regulated upon AR induction in response to exogenous auxin treatment, which seems to reprogram the cambium specific pathway to connect AR vascular tissue to the tap root^26^.

Finally, at the third time-point, 72 h after bending application, when protrusion of SLR was anatomically observed, *WOX5a* and *WOX11/12b* genes reached their maximum expression. While these genes have previously been reported to be expressed at different stages of AR formation^10,11,14^, in our case their expression was found to be much more specific at the later stage of root development.

The increased expression of the *WOX* genes analyzed in this paper, although it was not localized, might be related to the presence of several quiescent centers in the cambial zone of new root primordia strongly suggesting the involvement of these genes in SLR formation and development. However, further conclusive experiments are required to support this hypothesis.

Lateral roots produced by primary tissues differ from new SLRs, since during the formation of secondary tissues all primary tissues (including the pericycle) external to the vascular cylinder disappear, and the onset of both the vascular and cork cambia occurs^29–31^. SLRs play a pivotal role in higher plants, since they are produced in response to changes in environmental conditions^19,20^. However, despite the important functions played by SLRs (e.g. enhancement of plant anchorage to the soil), there is no agreement yet on the tissue source and molecular mechanisms involved in their formation. Our results support the hypothesis of a possible involvement of different *WOX* genes in SLR formation in Poplar, albeit their precise role remains to be determined. Moreover, our study has enabled us to collect evidence for a *WOX* genes regulation similar to that occurring in adventitious and lateral root initiation in primary tissues. Therefore, such studies as the one presented here become fundamental in efforts to enhance woody plant growth performance and stability by altering their root architecture and, thus, enlarging the horizons of agricultural and forestry practices.

## Conclusions

Overall, the conducted study highlights the expression of all *WOX* genes analyzed for the time points considered during SRL formation in *P. nigra*, triggered by the application of bending. These findings are in accordance with anatomical observations, which indicated that new lateral roots initiate from the cambial zone. Together, these results suggest that mechanical stress, determined by the application of bending, might activate molecular mechanisms in the bending zone leading to the expression of multiple *WOX* genes that control SLR formation in Poplar.

## Materials and methods

### Plant material and bending treatment

Anatomical analysis, aimed at detecting the development of a secondary structure along the taproot axis, was performed on 10 sixty-days-old *Populus nigra* (L.) seedlings. Subsequently, to stimulate the emission of new lateral roots (LRs) from a specific zone of the taproot, the portion characterized by a secondary structure was tied around a right angle-curved steel net to simulate mechanical perturbation^23,32–34^.

The bending sector (3 cm above and 3 cm below the point of maximum root bending) of the taproot was sampled from 15 poplar seedlings before bending application (time 0), as well as after 24, 48, and 72 h for a total number of 60 seedlings. At each time point, the whole bending sector was analyzed under a stereomicroscope (NIKON SMZ 800 N) to locate swellings, bumps or apices of a new LR. The sector of the taproot where new LR formation occurred, including the cambial zone and part of the xylem, was cut out, snap-frozen in liquid N_2_, and stored at − 80 °C until processed. Three independent biological replicates were tested.

Anatomical analysis was performed in accordance with De Zio et al.^33^. Briefly, 15 root samples for each time point were fixed, dehydrated and embedded, and then, cross sectioned (12 μm thick), stained and photographed using an OLYMPUS BX63 light microscope equipped with an OLYMPUS DP72 camera.

For molecular analysis, 70–100 mg of tissue powder obtained from root pieces of control and bent taproots were used separately for total RNA extraction. Total RNA was extracted using the mirPremier microRNA isolation kit (SIGMA-ALDRICH) according to the manufacturer’s instructions. Subsequently, total extracted RNA was treated with DNAse, measured by a spectrophotometer and used for the synthesis of 2 μg of cDNA employing the ImProm-II reverse transcription system kit (Promega). Primers for six *PnWOX* genes (*PnWOX4a*, *PnWOX4b*, *PnWOX5a*, *PnWOX5b*, *PnWOX11/12a*, and *PnWOX11/12b*) were chosen according to primers published for *Populus tomentosa*^10,11^. Real-time PCR was performed in the presence of SYBR Green Supermix (Bio-Rad), using the CFX Connect real-time PCR detection system and Maestro software (Bio-Rad). Cyclophylin (CYP) was used as an endogenous reference to normalize the expression levels of genes in qPCR^34^. Three independent biological replicates were tested in a final volume of 15 μl. The 2^−ΔΔCT^ method^35^ was applied to obtain the relative quantification of *PnWOXs* expression at different time points.

Results were elaborated in SPSS (SPSS Inc. Released 2009. PASW Statistics for Windows, Version 18.0. Chicago: SPSS Inc.). The Bonferroni test was applied to determine the statistical significance (*P* < 0.05) of differences in gene expression among the four time points.
